# Ex Vivo Study on the Antioxidant Activity of a Winemaking By-Product Polyphenolic Extract (Taurisolo^®^) on Human Neutrophils

**DOI:** 10.3390/antiox10071009

**Published:** 2021-06-23

**Authors:** Giuseppe Annunziata, Xavier Capó, Maria Magdalena Quetglas-Llabrés, Margalida Monserrat-Mesquida, Silvia Tejada, Josep A. Tur, Roberto Ciampaglia, Fabrizia Guerra, Maria Maisto, Gian Carlo Tenore, Ettore Novellino, Antoni Sureda

**Affiliations:** 1 NutraPharmaLabs, Department of Pharmacy, University of Naples Federico II, Via Domenico Montesano 49, 80131 Naples, Italy; giuseppe.annunziata@unina.it (G.A.); roberto.ciampaglia@unina.it (R.C.); fabrizia.guerra@unina.it (F.G.); maria.maisto@unina.it (M.M.); 2 Research Group in Community Nutrition and Oxidative Stress and Health Research Institute of the Balearic Islands (IdISBa), University of Balearic Islands-IUNICS, E-07122 Palma de Mallorca, Spain; xavier.capo@uib.es (X.C.); m.quetglas@uib.es (M.M.Q.-L.); margalida.monserrat@uib.es (M.M.-M.); silvia.tejada@uib.es (S.T.); pep.tur@uib.es (J.A.T.); antoni.sureda@uib.es (A.S.); 3 CIBEROBN (Physiopathology of Obesity and Nutrition), Instituto de Salud Carlos III, 28029 Madrid, Spain; 4 Laboratory of Neurophysiology, Biology Department and Health Research Institute of the Balearic Islands (IdISBa), University of Balearic Islands, E-07122 Palma de Mallorca, Spain; 5 NGN Healthcare—New Generation Nutraceuticals s.r.l., Torrette Via Nazionale 207, 83013 Mercogliano, Italy; ngnhealthcare@gmail.com

**Keywords:** oxidative stress, agri-food by-products, polyphenols, nutraceutical, grape, blood cells

## Abstract

Oxidative stress (OxS) has been linked to several chronic diseases and is recognized to have both major causes and consequences. The use of antioxidant-based nutraceuticals has been licensed as an optimal tool for management of OxS-related diseases. Currently, great interest is focused on the valorization of agri-food by-products as a source of bioactive compounds, including polyphenols. In this sense, we evaluated the efficacy of a novel nutraceutical formulation based on polyphenolic extract from *Aglianico* cultivar grape pomace (registered as Taurisolo^®^). In particular, we tested both native and in vitro gastrointestinal digested forms. The two extracts have been used to treat ex vivo neutrophils from subjects with metabolic syndrome, reporting a marked antioxidant activity of Taurisolo^®^, as shown by its ability to significantly reduce both the levels of reactive oxygen species (ROS) and the activities of catalase and myeloperoxidase in the cell medium after stimulation of neutrophils with phorbol 12-myristate 13-acetate (PMA). Interestingly, we observed an increase in intracellular enzymatic activities in PMA-treated cells, suggesting that Taurisolo^®^ polyphenols might be able to activate nuclear factors, up-regulating the expression of this target antioxidant gene. In addition, Taurisolo^®^ reversed the increase in malondialdehyde induced by PMA; reduced the expression of pro-inflammatory genes such as cyclooxygenase 2 (COX-2), tumor necrosis factor alpha (TNFα) and myeloperoxidase (MPO); and induced the expression of the anti-inflammatory cytokine IL-10. Overall, these results suggest the efficacy of Taurisolo^®^ in contrasting the OxS at blood level, providing evidence for its therapeutic potential in the management of OxS-related pathological conditions in humans.

## 1. Introduction

The term “oxidative stress” (OxS) refers to an imbalance between production and elimination of oxidants, mainly free radicals, in favor of their generation, leading to alterations of redox signaling and control [1]. Among the main oxidant agents, there are reactive oxygen species (ROS) (including ^•^OH, O_2_^•−^, HO_2_^•^, and ROO^•^) and reactive nitrogen species (RNS) (including NO^•^ and ^•^ONOO) [2]. Physiologically, ROS/RNS serve as signaling molecules involved in important biological processes, including cell proliferation, programmed cell death, and gene expression [3,4]. However, the chronic and progressive accumulation of both ROS and RNS damages biological macromolecules, such as sugar, lipids, proteins, and nucleic acids, leading to both pathophysiological alterations and accelerating ageing, finally culminating in the development of several diseases [5], including cardiovascular diseases, cancer, diabetes, and neurodegenerative disorders [6,7]. The production of reactive species can derive from aerobic metabolism itself, but also from immune cells as an element of their antimicrobial activity through NADPH oxidase and myeloperoxidase (MPO) [8,9]. Notably, when the increase of ROS/RNS is within certain limits, the organism is able, by the biology resolution pathway, to deactivate them in order to counteract the OxS-activating antioxidant defense systems, including specific enzymes such as catalase (CAT) and superoxide dismutase (SOD) that act as free radical scavengers [10]. Nevertheless, if OxS is excessive or is maintained over time, and endogenous antioxidant defense is not able to counteract this situation, it can lead to oxidative damage. In order to avoid damage to biomolecules, the use of exogenous molecules in the form of antioxidant supplements may represent a useful tool for prevention and management of OxS-related diseases. In this sense, polyphenols have been licensed as promising candidates. As antioxidant molecules with peculiar chemical features, indeed, polyphenols are able to directly inhibit the generation of ROS or promote their reduction; in addition, polyphenols can also modulate the activities of endogenous antioxidant defenses [11].

Over the years, this promising evidence drove the research in nutraceutical field toward identification of polyphenol-rich food matrices for the formulation of supplements aimed to counteract the OxS. Interestingly, in the last decade a growing interested focused on reutilization and valorization of by-products produced by the agri-food industry as a potential raw material for formulation of nutraceuticals [12].

Agri-food industry by-products represent a relevant issue in terms of economic and environmental impact. About 60% of raw materials used by the food-industry, indeed, constitutes part of the waste material, mainly destined for animal feed, production of fertilizes, composting, and biogas [12]. Evidence highlights the importance of the so-called *agri-food by products* reutilization [13,14,15], underlying further applications, ranging from cosmetic to the pharmaceutical industry [12], with particular interest in the nutraceutical sector [16].

It is well known that *agri-food industry by-products* represent an important source of bioactive compounds [16], including organic acids, pigments, fiber, essential oils, vitamins, and antioxidants [17], occasionally present in higher amounts than in the edible part of the food [16]. In vivo studies demonstrated that, due to the high content of polyphenols, winemaking *by-products* exert greater beneficial effects on human health than the red wine [18]. Indeed, winemaking *by-products*, commonly called grape pomace, are extraordinarily rich in bioactive compounds, mainly polyphenols [19,20,21], making them a useful source with a high nutraceutical potential.

In this sense, we studied the antioxidant activity of a novel nutraceutical formulation based on a polyphenolic extract from *Aglianico* cultivar grape pomace (registered as Taurisolo^®^) on human blood cells. Taurisolo^®^ was designed as a grape polyphenol extract microencapsulated in maltodextrins, in order to improve the bioaccessibility of the polyphenols. In addition, the effects of the extract after in vitro gastrointestinal digestion have been investigated to mimic, as closely as possible, the human physiology.

## 2. Materials and Methods

### 2.1. Reagents

All chemicals and reagents used were either analytical or HPLC-grade reagents. The water was treated in a Milli-Q water purification system (Millipore, Bedford, MA, USA) before use. Chemicals and reagents used to simulate the gastrointestinal digestion: potassium chloride (KCl); potassium thiocyanate (KSCN); monosodium phosphate (NaH_2_PO_4_); sodium sulphate (Na_2_SO_4_); sodium chloride (NaCl); sodium bicarbonate (NaHCO_3_); hydrochloric acid (HCl); and also the enzymes pepsin (≥250 U/mg solid) from porcine gastric mucosa and pancreatin (4 × USP) from porcine pancreas were purchased from Merck Life Science (Milan, Italy). 2,2-diphenyl-1-picrylhydrazyl (DPPH) and 2,20-azinobis(3-ethylbenzotiazoline-6- sulfonate) (ABTS), Hank’s balanced salt, 2,7-dichlorofluorescin-diacetate (DCFH-DA) 1-(4,5-dimethylthiazol-2-yl) -3, 5-diphenylformazan (MTT), dimethyl sulfoxide (DMSO), RPMI 1640 culture media, phorbol myristate acetate (PMA), tripure^®^, xanthine, xanthine oxidase, cytochrome C, hydrogen peroxide, guaiacol, methanol, acetonitrile, and n-methyl-2-phenylindole were purchased from Merck Life Science (Madrid, Spain). Retro transcription and real time PCR reagents were purchased from Roche Diagnostics (Mannheim, Germany).

### 2.2. Taurisolo^®^ Supplement Preparation

To produce the nutraceutical supplement Taurisolo^®^, polyphenols were extracted from *Aglianico* cultivar grapes, collected during the autumn 2018 harvest. The first pilot production of the polyphenol extract was optimized at the *NutraPharmaLabs* of the Department of Pharmacy, University of Naples Federico II (Naples, Italy); subsequently, the MB-Med Company (Turin, Italy) accomplished the large-scale production. Briefly, grapes were extracted with hot water (50 °C); the obtained solution was filtrated, concentrated, and underwent a spray-drying with maltodextrins in a range of 5–15%, obtaining a fine microencapsulated powder. The polyphenol profile of Taurisolo^®^ was evaluated by High-Performance Liquid Chromatography-diode array detector (HPLC-DAD, Jasco Inc., Easton, MD, USA) analysis using the method described by Giusti et al., 2017 [22] and previously reported [23].

### 2.3. In Vitro Gastrointestinal Digestion

In order to mimic the human physiology, since our experiments were performed on blood cells (as detailed below), acid-resistant capsules containing 800 mg Taurisolo^®^ have undergone in vitro GI digestion protocols, following the procedure described in our previous studies [24,25]. The protocol used is detailed in Supplementary Materials.

### 2.4. Antioxidant Activity of Taurisolo^®^

The total antioxidant capacity was measured using the ferric reducing antioxidant power (FRAP) assay, following the method described by Benzie and Strain [26]. Also, the antioxidant activity of both native and digested Taurisolo^®^ was evaluated using the DPPH and the ABTS assays. The protocols used are detailed in Supplementary Materials.

### 2.5. Cell Isolation and Cell Viability Test

Blood samples were collected at 08:00 from 12 h-fasting adults with metabolic syndrome (MetS), in suitable vacutainers with EDTA as an anticoagulant. Neutrophil fraction was purified following an adaptation of the method described by Bøyum [27,28]. The protocol used is detailed in Supplementary material.

Cell viability was evaluated by a MTT assay, as previously described [29].

### 2.6. Cell Treatment and Experimental Design

P(*n* = 14) were cultured with RPMI 1640 culture medium containing 2 mM L-glutamine and divided into six aliquots: (i) control group (Ctr; neutrophils treated only with culture medium), (ii) control + Taurisolo^®^ group (Taurisolo^®^; neutrophils treated with culture medium in addition to 1 mg/mL Taurisolo^®^), (iii) control + digested-Taurisolo^®^ group (digested-Taurisolo^®^; neutrophils treated with culture medium in addition to 1 mg/mL Taurisolo^®^ underwent in vitro gastrointestinal digestion), (iv) phorbol 12-myristate 13-acetate (PMA) group (PMA; neutrophils treated with culture medium in addition to 5 µg/mL PMA), (v) PMA + Taurisolo^®^ group (PMA + Taurisolo^®^; neutrophils treated with culture medium in addition to 5 µg/mL PMA and 1 mg/mL Taurisolo^®^), and (vi) PMA + digested-Taurisolo^®^ group (PMA + digested-Taurisolo^®^; neutrophils treated with culture medium in addition to 5 µg/mL PMA and 1 mg/mL Taurisolo^®^ underwent in vitro gastrointestinal digestion). All neutrophil groups were incubated in polypropylene tubes at 37 °C for 2 h. Subsequently, the cells were pelleted by centrifugation (900× *g*, 5 min, 4 °C) and cell-free supernatants were stored at −80 °C until biochemical determinations. Incubations were performed in duplicate, one replicate was used for RNA extraction and the other to evaluate *OxS* markers; the determinations made in the cell-free supernatants will be considered as determinations in the extracellular media. In samples for OxS markers, neutrophils were resuspended with 2 mL of PBS and one aliquot (1 mL) was centrifuged at 900× *g*, 5 min, 4 °C, and the precipitate containing the neutrophils was lysed with distilled water and stored at −80 °C; determinations performed in the neutrophil lysates will be considered as determinations in the intracellular media.

### 2.7. Hydrogen Peroxide Production

H_2_O_2_ production was measured in neutrophil samples using 2,7-dichlorofluorescin-diacetate (DCFH-DA) as an indicator. A stock solution of DCFHDA (1 mg/mL) in ethanol was prepared and stored at 20 °C until analysis. DCFH-DA (30 µg/mL) in PBS was added to a 96-well microplate containing 50 µL of neutrophil suspension from each one of the six groups. The fluorescence (Ex, 480 nm; Em 530 nm) was recorded at 37° C for 1 h in FL 9800 Microplate Fluorescence Reader (Bio-tek Instruments, Inc.).

### 2.8. Oxidative Stress-Related Enzyme Activity

The activities of CAT and MPO were determined both in extracellular and intracellular media. Both enzyme activities were determined with a Shimadzu UV-2100 spectrophotometer at 37 °C. MPO activity was measured by guaiacol oxidation [30]. The reaction mixture contained sodium phosphate buffer pH 7 and 13.5 mM guaiacol. The reaction was initiated by adding 300 mM H_2_O_2_, and changes at 470 nm were monitored. CAT activity was measured by the spectrophotometric method of Aebi [31] based on following the decomposition of H_2_O_2_ at 240 nm.

### 2.9. Malondialdehyde Assay

Malondialdehyde (MDA) as a marker of lipid peroxidation was analyzed using a colorimetric assay based, as previously reported [32]. The protocol used is detailed in Supplementary Materials.

### 2.10. Gene Expression

At the end of incubation, neutrophils were centrifugated at 900× *g*, 5 min, 4 °C and supernatants were discarded. One milliliter of Tripure^®^ was added to each sample to extract and purify RNA. RNA extraction was performed following manufacturer instructions. The protocol used is detailed in Supplementary Materials.

Cyclooxygenase (COX)-2, interleukin (IL)-10, tumor necrosis factor (TNF)α, and MPO expression were determined by multiplex real time rtPCR using human 18S rRNA as invariant reference. The primers and amplification conditions used are listed in Table 1.

### 2.11. Statistical Analysis

Statistical analysis was carried out using the Statistical Package for Social Sciences (SPSS v.25.0 for Windows). Results are presented as mean ± SEM, and *p* < 0.05 was considered statistically significant. A Shapiro–Wilk test was performed to assess the normal distribution of the data. When the data were normally distributed, statistical significance was assessed by one-way analysis of variance (ANOVA) depending on the sample analyzed.

## 3. Results

### 3.1. Chemical Profile of Native and Digested-Taurisolo^®^

The polyphenol profile of Taurisolo^®^ before and after in vitro simulated GI digestion was evaluated by HPLC-DAD analysis. The main polyphenols are reported in Table 2.

### 3.2. Characteristics of Study Participants

Seventeen subjects with diagnosis of MetS were enrolled. Overall, subjects were middle-aged (63.4 ± 10.9 years) and overweight-obese (body mass index: 31.6 ± 3.14 kg/m^2^). Anthropometric characteristics and metabolic and hematological parameters are reported in Table 3.

### 3.3. Antioxidant Activity of Taurisolo^®^

The antioxidant activity of both digested and non-digested Taurisolo^®^ was evaluated in vitro through the FRAP, DPPH, and ABTS assay. As reported in Table 4, native Taurisolo^®^ presents a relatively high antioxidant capacity that is slightly decreased after gastrointestinal digestion (~30%).

### 3.4. Cytotoxicity of Taurisolo^®^

**Figure 1:**
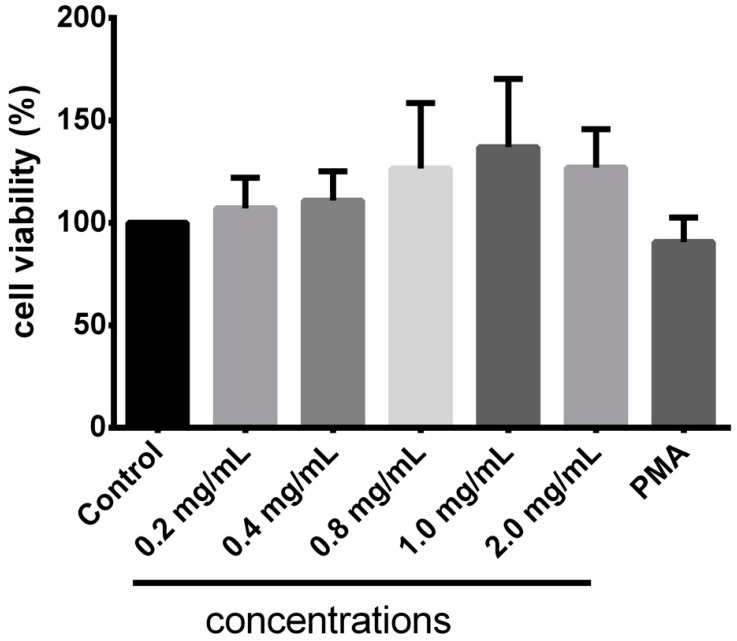
Cell viability test. Results are expressed as mean ± SD of three repetitions.

The cell viability test revealed that Taurisolo^®^ did not exert any cytotoxic effect at concentrations ranging from 0.2 mg/mL to 2.0 mg/mL (Figure 1). The apparently dose-dependent increased cell viability might be explained by the 570 nm absorbance of polyphenols contained in our extract.

### 3.5. Taurisolo^®^ Reduces the Levels of ROS

**Figure 2:**
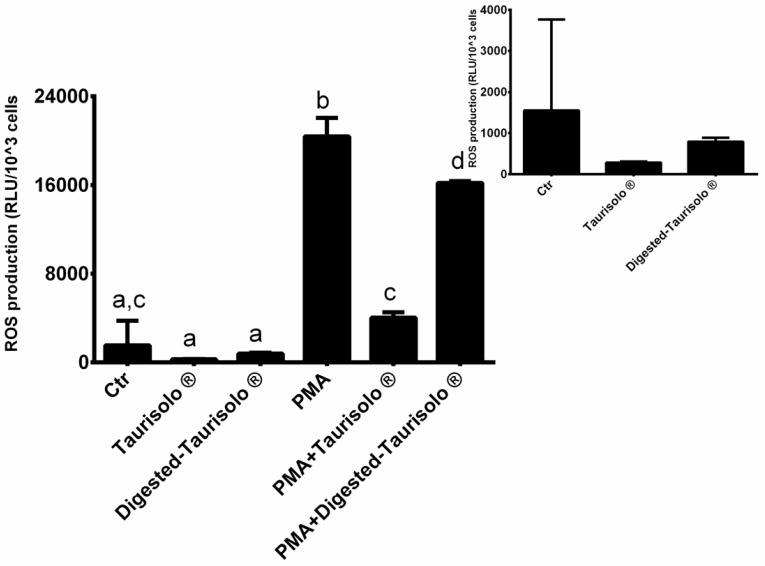
ROS levels monitored evaluating the production of hydrogen peroxide. Results are expressed as mean ± SEM, and *p* < 0.05 was considered statistically significant. ANOVA one-way analysis. Different letters reveal significant differences.

The levels of ROS were indirectly monitored via evaluation of hydrogen peroxide production in neutrophils incubated with/without Taurisolo^®^ (both native and digested) in the presence or absence of PMA (Figure 2). As expected, PMA significantly increased the ROS production compared to control (+1214.50%). On the contrary, the incubation with Taurisolo^®^ and digested-Taurisolo^®^ significantly reduced the ROS production in the absence as well as in the presence of PMA (Taurisolo^®^: −81.60% compared to Ctr; PMA + Taurisolo^®^: −80.23% compared to PMA; digested-Taurisolo^®^: −48.86% compared to Ctr; PMA + digested-Taurisolo^®^: −20.55% compared to PMA).

### 3.6. Taurisolo^®^ Modulates the Enzymatic Activities of CAT and MPO

**Figure 3:**
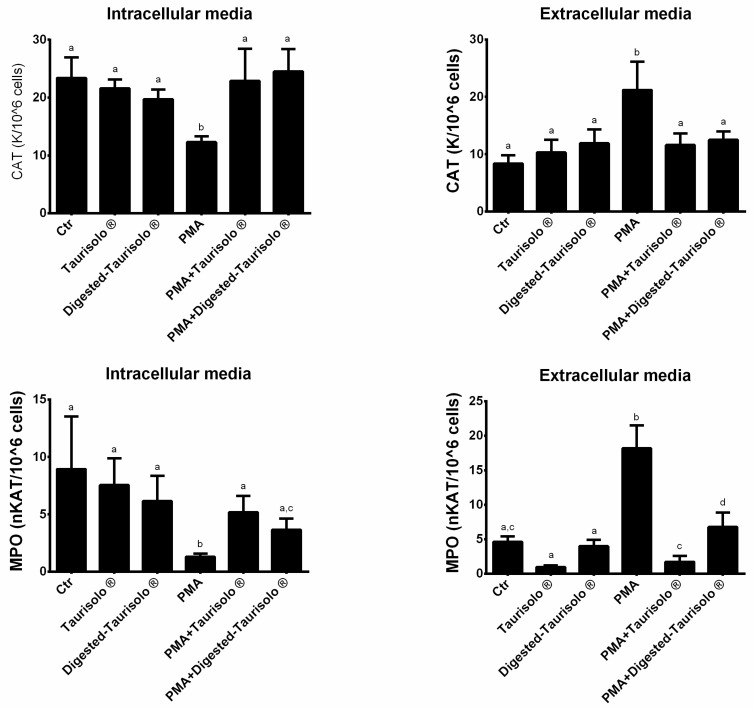
Enzymatic activities of CAT and MPO. Results are expressed mean ± SEM and *p* < 0.05 was considered statistically significant. ANOVA one-way analysis. Different letters reveal significant differences.

The enzymatic activities of CAT and MPO were determined both in extracellular and intracellular with spectrophotometric assays (Figure 3). As expected, in extracellular media, PMA significantly increased the activity of both the enzymes when compared to control (+153.28% and +293.94%, CAT and MPO, respectively, *p* < 0.001 for all). On the contrary, the incubation with Taurisolo^®^ and digested-Taurisolo^®^ significantly reduced the enzymatic activities in the presence of PMA (CAT = PMA + Taurisolo^®^: −45.28% compared to PMA, PMA + digested-Taurisolo^®^: −41.03% compared to PMA; MPO = PMA + Taurisolo^®^: −90.60% compared to PMA, PMA + digested-Taurisolo^®^: −62.80% compared to PMA). On the other hand, in intracellular media, the trend was diametrically opposed. As shown, indeed, PMA significantly reduced the enzymatic activities of CAT and MPO (−47.43% and −85.46%, respectively), while the incubation with Taurisolo^®^ and digested-Taurisolo^®^ caused their increase (CAT = PMA + Taurisolo^®^: +86.17% compared to PMA, PMA + digested-Taurisolo^®^: +99.19% compared to PMA; MPO = PMA + Taurisolo^®^: +298.46% compared to PMA, PMA + digested-Taurisolo^®^: 181.54% compared to PMA).

### 3.7. Taurisolo^®^ Reduces the MDA Levels

**Figure 4:**
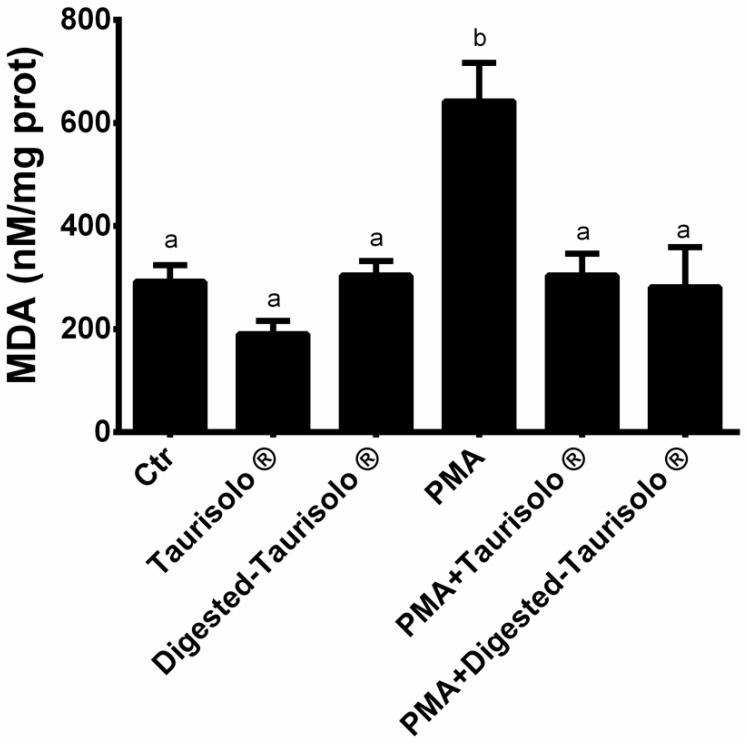
Levels of MDA. Results are expressed as mean ± SEM, and *p* < 0.05 was considered statistically significant. ANOVA one-way analysis. Different letters reveal significant differences.

We evaluated the ability of Taurisolo^®^ to prevent oxidative damage; we monitored the levels of MDA as lipid peroxidation markers. As shown in Figure 4, PMA significantly increased MDA levels (+119.86% compared to control), while incubation with Taurisolo^®^ and digested-Taurisolo^®^ significantly reduced (PMA+ Taurisolo^®^: −52.65% compared to PMA; PMA + digested-Taurisolo^®^: −56.23% compared to PMA).

### 3.8. Taurisolo^®^ Modulates the Expression of OxS- and Inflammation-Related Genes

The effect of Taurisolo^®^ treatment on expression of OxS- and inflammation-related genes was evaluated only in neutrophils cultured with digested-Taurisolo^®^ since maltodextrins present in native Taurisolo^®^ caused such interferences during the Real-Time PCR reaction [33,34]. Moreover, digested-Taurisolo^®^, as in vitro gastrointestinal digestion-derived duodenal phase, represents the polyphenol fraction potentially absorbed in vivo, thus reaching the bloodstreams. In this sense, evaluation of the gene expression modulating effects of digested-Taurisolo^®^ on blood cells was more appropriated. As shown in Table 5, we observed that digested-Taurisolo^®^ efficiently acts in the modulation of targeted genes, in particular down-regulating OxS- and inflammation-related genes and up-regulating anti-inflammatory gene.

## 4. Discussion

In the present study, we demonstrated the antioxidant activity of a novel nutraceutical formulation, registered as Taurisolo^®^, obtained from *Aglianico* cultivar winemaking by-products, on human immune cells. More specifically, we demonstrated the ability of Taurisolo^®^ to significantly reduce the levels of ROS and the activities of antioxidant enzymes in extracellular medium, and to enhance the intracellular antioxidant enzymatic systems in neutrophils. These observed results are in line with the available literature reporting the antioxidant potential of grape polyphenols. In particular, a recent study demonstrated the ROS-reducing effect of intravenous and oral administration of Taurisolo^®^ in a rat model of brain microvascular alteration induced by diminished cerebral blood flow and subsequent blood flow restoration [35]. Similarly, we also demonstrated in humans that chronic oral administration of Taurisolo^®^ significantly reduced the levels of OxS- and cardiovascular-related biomarkers, including trimethylamine N-oxide and oxidized low density lipoprotein cholesterol in healthy [25] and overweight/obese subjects [36].

These promising results led us to investigate the potential mechanisms of action of Taurisolo^®^ for its antioxidant effects and its role at the blood level. For this reason, we decided to test the efficacy of our extract in human neutrophils from subjects with MetS, in which both redox unbalance and inflammation affecting the cellular response and immune cells have been clearly demonstrated [37,38,39,40]. Particularly, a typical feature in this class of subjects is the so-defined chronic low-grade inflammation, also characterized by increased circulating levels of pro-inflammatory cytokines and biomarkers of oxidative stress [41,42,43]. In this sense, since neutrophils are implicated in various anti-pathogen functions, including OxS and inflammation; we decided to use this cellular model to assess the efficacy of Taurisolo^®^. Also, in order to mimic as close as possible the human physiology, we tested Taurisolo^®^ before and after in vitro-simulated gastrointestinal digestion. More specifically, after this in vitro protocol, the fraction obtained from the intestinal phase (indicated as digested-Taurisolo^®^) was considered as the fraction potentially absorbed in vivo after oral administration and thus reaching the bloodstream.

As previously reported, Taurisolo^®^ is a miscellanea of polyphenols, which includes about 7 mg/g of targeted polyphenolic compounds [23] with demonstrated antioxidant activity both in vitro and in vivo [23,25,32,36,44]. This articulated polyphenol profile is responsible for the OxS-contrasting effect of Taurisolo^®^, as shown by the FRAP assay and the free radical scavenging activity (DPPH assay, ABTS assay and reduction of ROS levels). Due to their chemistry, indeed, polyphenols are directly involved in reactions with free radicals, including hydroxyl, superoxide, nitric oxide, alkoxyl, and peroxyl radicals. In particular, through their benzene ring-bound hydroxyl groups, polyphenols are responsible for an electron transfer on ROS molecules, which stabilizes the reactive species [45,46]; additionally, these reactions generate phenoxyl radicals that react with a second radical, leading to the formation of a stable quinine structure [46]. Moreover, polyphenols are also able to chelate iron and copper ions, preventing their participation in free radical formation reactions [11], and thus, contrasting the ROS production. These two mechanisms were previously reported to explain the polyphenol ability to scavenge ROS and inhibit their generation [11]. In our experiments, both digested and non-digested Taurisolo^®^ treatment were shown to reduce the ROS levels in control cells, suggesting their ability to contrast the free radical accumulation also in basal conditions. However, to recreate a pathological condition, we stimulated neutrophils with PMA to induce the degranulation and oxidative burst with consequent large production of ROS [47]. These processes were evidenced by the increase in MPO in the extracellular medium and in ROS production, accompanied by a MPO decrease at the intracellular level. Altogether, they can contribute to the pro-oxidant and subchronic inflammation state characteristic of patients with metabolic syndrome [48]. Also, in the presence of PMA, both digested and non-digested Taurisolo^®^ treatment drastically reduced the levels of ROS in the extracellular medium, suggesting their free radical scavenging potential both in normal and pathological conditions.

The increased levels of free radicals in the cellular medium related to NADPH oxidase and MPO may be considered as the main actor for the activation of cell responses, resulting in the release of antioxidant enzymes aimed atcontrasting the ROS accumulation and cellular damage, including CAT, which decomposes H_2_O_2_ into H_2_O before H_2_O_2_ reacts with metal ions generating hydroxyl radicals [10]. This mechanism is supported by the observed marked increase in the enzymatic activities of CAT in the extracellular medium after stimulation with PMA in parallel with MPO [47]. However, after treatment with both digested and non-digested Taurisolo^®^, we registered a marked decrease of the activities of these two enzymes. It can be speculated, thus, that the ROS-reducing effect of Taurisolo^®^-polyphenols might be responsible for the reduced MPO and CAT enzymatic activities with or without the PMA stimulation, suggesting the ability of antioxidants to contrast the global oxidative stress in the culture medium that results in reducing the need to activate the intracellular defenses for neutralization of the dangerous external environment.

Interestingly, when we analyzed the enzymatic activities of CAT and MPO in the intracellular medium, we observed an inverse trend. In particular, in cells treated with PMA, the activities of CAT and MPO were reduced, while they were increased when cells were treated with both digested and non-digested Taurisolo^®^. This effect may be explained by the ability of polyphenols to modulate the endogenous antioxidant defense [11]. More specifically, there is evidence that polyphenols can increase the activities of antioxidant enzymes, up-regulating the expression of related genes via activation of specific nuclear signaling pathways [11,49]. It was reported, indeed, that grape-deriving polyphenols activate nuclear factors, including nuclear factor-erythroid 2-related factor 2 (Nrf2) and forkhead box 0 (FOX0) and proliferator-activated receptor gamma coactivator 1α (PGC-1 α), causing their nuclear translocation [10]. This, in turn, results in enhancement of the expression of responding genes encoding for antioxidant enzymes. In contrast, there is evidence recognizing free radicals as stimuli for activation of the nuclear factor kappa-light-chain-enhancer of activated B cells (NF-κB), in which nuclear translocation promotes the expression of OxS- and inflammation-related genes. Interestingly, grape polyphenols are capable of suppressing the NF-κB pathways, suggesting a further antioxidant and anti-inflammatory mechanism [10]. These previously reported mechanisms may be used to explain the observed Taurisolo^®^-induced increase of the antioxidant enzyme activities in intracellular medium either in basal condition or under PMA stimulation.

The different trends between the antioxidant enzymatic activities observed in extracellular and intracellular media led us to speculate a dual efficacy of Taurisolo^®^ in contrasting OxS: On one hand, the ROS-reducing effect that results in improving the redox status of the external environment, reducing the needs of the cells to respond with their endogenous antioxidant systems; on the other hand, the intrinsic activation of the antioxidant defenses, probably via modulation of the related gene expression. This is corroborated by our results on the effects of digested-Taurisolo^®^ in down-regulating the expression of OxS- and inflammation-related genes (COX-2 and TNFα) and up-regulating that of anti-inflammatory gene (IL-10). Interestingly, we observed that in basal conditions (that is in absence of stimulation with PMA), digested-Taurisolo^®^ treatment causes an upregulation of the COX-2 gene. This might be explained with the hormesis theory, suggesting that biological systems are able to activate bidirectional responses to various harmful stimuli [29,50]. More specifically, according to this theory, such stimuli are able to induce positive or negative effects based on the dosage. For example, ROS are capable to (i) stimulate specific signaling pathways, receptors, or antioxidant enzymes at low doses; and (ii) inhibit antioxidant enzyme or activate pro-inflammatory pathways at high doses [29,50,51,52,53,54]. It appears clear, thus, that cells can react to such stimuli with a double directionality, following a bell-shaped fashion, on the base of stimulus intensity [29]. Remarkably, this theory can be also applied to antioxidants. In particular, it was reported that these bioactive compounds can induce hormetic responses, suggesting a possible explanation for the observed upregulation of COX-2. The antioxidant-dependent hormetic response is not able to induce any tissue damage, but it serves as an intrinsic stimulus for activation of endogenous defense systems [55,56,57]. However, considering both the observed down-regulation of MPO gene and the absence of other OxS and inflammation-related genes, further investigation of the gene expression modulating effect of Taurisolo^®^ is needed to confirm our preliminary results.

Notably, the high antioxidant activity of Taurisolo^®^ is also demonstrated by its ability to reduce the MDA levels, suggesting the role played in preventing the oxidative damage at cell membrane level. More specifically, lipid peroxidation is promoted by both ROS and RSN [10] via chain-reactions, culminating with the production of reactive aldehydes, including MDA [58,59,60]. These molecules are able to penetrate the biological membranes attacking biomolecules, thus causing their biological and biochemical alteration [61,62,63].

## 5. Conclusions

**Figure 5:**
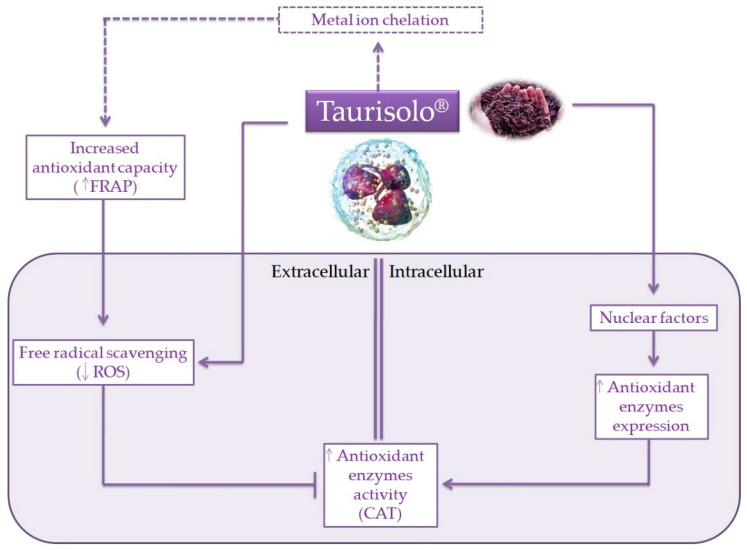
Schematic representation of the potential mechanisms of action of Taurisolo^®^. As a polyphenol-rich extract, Taurisolo^®^ has a marked antioxidant capacity that, in addition to the metal ion chelation activity, acts as a free radical scavenging agent, reducing the levels of ROS in the extracellular medium. This improvement of the redox status of the external environment results in reduced needs of the cell to activate their endogenous defenses, releasing antioxidant enzymes. At the nuclear level, indeed, Taurisolo^®^ polyphenols are able to interact with nuclear factors, resulting in enhancing the expression of antioxidant genes. ____ indicates the mechanisms directly observed; ----- indicates the mechanisms/pathways not directly observed, but reported in literature.

In summary, with the present ex vivo study we demonstrated the elevated antioxidant potential of Taurisolo^®^, a novel nutraceutical formulation based on a polyphenolic extract obtained from *Aglianico* cultivar winemaking *by-products*, on human neutrophils from subjects with MetS. As shown in Figure 5, due to its antioxidant capacity, Taurisolo^®^ firstly acts as a ROS scavenger agent, reducing their levels in the extracellular medium. This, in turn, may be responsible for reduced activities of antioxidant enzymes in the same medium, as a result of improved redox status of the external environment with consequent reduction of the cell response needed. In addition, the observed increase of antioxidant enzyme activities in the intracellular medium might reflect the ability of Taurisolo^®^ polyphenols to up-regulate their gene expression. Overall, data herein presented suggest the relevant potential of this *agri-food by-product* as a source of bioactive compounds for formulation of nutraceutical supplements aimed at managing OxS-related diseases. To the best of our knowledge, this is the first study demonstrating these beneficial activities of grape pomace polyphenols on human blood cells, representing further strong evidence about the importance and usefulness of *by-product* reutilization in pharmaceutical industry. Our results, in addition, might drive scientific research toward further in vivo studies investigating the role of such nutraceuticals in prevention and management of OxS-related diseases.

## Figures and Tables

**Table 1:** Primers sequences and Real-Time PCR conditions.

Gene	Accession Number	Sequence	Temperature (°C)
**COX-2**	NM_000963	Fw: **5′**-TTGCCTGGCAGGGTTGCTGGTGGTA-3′	95 °C 10 Seg 63 °C 10 Seg 72 °C 15 Seg
		Rev: **5′**-CATCTGCCTGCTCTGGTCAATGGAA-3′	
**IL-10**	NM_000572	Fw: **5′**-AGAACCTGAAGACCCTCAGGC-3′	95 °C 10 Seg 58 °C 10 Seg 72 °C 15 Seg
		Rev: **5′**-CCACGGCCTTGCTCTTGTT-3′	
**TNFα**	NM_000594	Fw: **5′**-CCCAGGCAGTCAGATCATCTTCTCGGAA-3′	95 °C 10 Seg 63 °C 10 Seg 72 °C 15 Seg
		Rev: **5′**-CTGGTTATCTCTCAGCTCCACGCCATT-3′	
**MPO**	NM_000250	Fw: **5′**-TGAACATGGGGAGTGTTTCA-3′	95 °C 10 Seg 62 °C 10 Seg 72 °C 15 Seg
		Rev: **5′**-CCAGCTCTGCTAACCAGGAC-3′	
**18S**	NR_003286	Fw: **5′**-ATG TGA AGT CAC TGT GCC AG-3′	95 °C 10 Seg 60 °C 10 Seg 72 °C 12 Seg
		Rev: **5′**-GTG TAA TCC GTC TCC ACA GA-3′	

Abbreviations: COX-2, cyclooxygenase-2; IL-10, interleukin-10; TNFα, tumor necrosis factor α; MPO, myeloperoxidase.

**Table 2:** High Performance Liquid Chromatography-diode-array detector (HPLC-DAD) analysis of the main polyphenols contained in Taurisolo^®^, before and after in vitro simulated gastrointestinal digestion. Values are expressed in µg/g Taurisolo^®^ ± standard deviation of three repetitions.

Compound	Mean Value (µg/g) ± SD	
	*Taurisolo^®^*	*Digested Taurisolo^®^*
Ferulic acid	14.59 ± 0.98	1.92 ± 0.11
Resveratrol	12.55 ± 0.02	0.245 ± 0.004
Caffeic acid	35.00 ± 3.00	n.d.
*p*-cumaric acid	122.75 ± 2.77	n.d
Rutin	98.81 ± 7.31	20.82 ± 0.20
Quercetin	135.41 ± 4.69	n.d.
Procyanidin B1 dimer	946.33 ± 55.20	116.72 ± 0.52
Procyanidin B2 dimer	645.89 ± 59.17	169.16 ± 0.77
Syringic acid	310.95 ± 0.01	122.57 ± 0.55
Epicatechin	1696.55 ± 109.60	1.38 ± 0.02
Gallic acid	199.46 ± 4.59	n.d.
Catechin	2499.04 ± 307.41	77.95 ± 0.29

**Table 3:** Characteristics of study participants.

Parameter	Mean ± SEM (*n* = 17)
Age (years)	63.4 ± 10.9
***Anthropometric characteristics***	
Weight (kg)	91.4 ± 17.4
Height (cm)	169.5 ± 12.1
BMI (kg/m^2^)	31.6 ± 3.14
***Metabolic parameters***	
Glucose (mg/dL)	103.2 ± 27.5
Hb1A (%)	5.92 ± 1.23
Triglycerides (mg/dL)	200.6 ± 31.7
HDL-cholesterol (mg/dL)	40.4 ± 8.57
LDL-cholesterol (mg/dL)	126.5 ± 28.9
Cholesterol total (mg/dL)	197.1 ± 40.3
Bilirubin (mg/dL)	0.683 ± 0.223
AST (U/L)	21.2 ± 4.02
ALT (U/L)	24.9 ± 9.96
GGT (U/L)	39.1 ± 31.4
PKC (mg/dL)	0.770 ±0.310
***Hematological parameters***	
Hematocrit (%)	46.0 ± 2.69
Erythrocytes (10^6^/mm^3^)	4.99 ± 0.374
Leukocytes (10^3^/mm^3^)	7.83 ± 1.63
Neutrophils (10^3^/mm^3^)	4.30 ± 1.39
Lymphocytes (10^3^/mm^3^)	2.58 ± 0.763
Basophils (10^3^/mm^3^)	0.063 ± 0.038
Monocytes (10^3^/mm^3^)	0.648 ± 0.189
Eosinophils (10^3^/mm^3^)	0.238 ± 0.084
Platelets (10^3^/mm^3^)	218.7 ± 62.5

**Table 4:** Antioxidant activity of Taurisolo^®^ evaluated by FRAP, DPPH, and ABTS assays. Values are expressed in mM TE ± SEM (FRAP) and mmol TE/g ± SEM (DPPH and ABTS) of three repetitions.

Sample	DPPH (mmol TE/g)	ABTS (mmol TE/g)	FRAP (mM TE)
**Taurisolo^®^**	3.67 ± 0.55	3.47 ± 0.11	0.705
**Digested-Taurisolo^®^**	2.55 ± 0.76	2.33 ± 0.66	0.530

**Table 5:** Effect of Taurisolo^®^ polyphenols on OxS- and inflammation-related gene expression.

	Control	Digested-Taurisolo^®^	PMA	PMA + Digested-Taurisolo^®^	ANOVA		
					T	A	TxA
COX-2	1.00 ± 0.28 ^a^	1.87 ± 0.33 ^a^	10.4 ± 4.59 ^b^	0.54 ± 0.12 ^a^			x
IL-10	1.00 ± 0.27	8.23 ± 2.34	5.25 ± 1.82	9.87 ± 4.19			
TNFα	1.00 ± 0.59 ^ab^	0.21 ± 0.04 ^a^	5.32 ± 1.17 ^c^	2.80 ± 0.86 ^b^	x	x	
MPO	1.00 ± 0.21 ^a^	0.45 ± 0.07 ^b^	1.25 ± 0.22 ^b^	0.41 ± 0.05 ^b^	x		

Results are expressed as mean ± SEM, and *p* < 0.05 was considered statistically significant. Statistical significance was calculated by two-way ANOVA analysis. Factor T (digested-Taurisolo^®^), Factor A (activation with PMA). Different letters reveal significant differences.

## Data Availability

The data used to support the findings of this study are included within the article.

## Supplementary Materials

The following are available online at https://www.mdpi.com/article/10.3390/antiox10071009/s1.
