# Supplementary material for: Ex Vivo Study on the Antioxidant Activity of a Winemaking By-Product Polyphenolic Extract (Taurisolo®) on Human Neutrophils
Source: Antioxidants (Basel). 2021 Jun 23;10(7):1009. doi: 10.3390/antiox10071009 (PMC8300751; doi:10.3390/antiox10071009)
Supplement: Supplementary file 1 [file antioxidants-10-01009-s001.zip › antioxidants-1248499-supplementary.pdf]

Supplementary Materials

In Vitro Gastrointestinal Digestion

Capsules were mixed to 6 mL of artificial saliva (components: KSCN (20 g/L), KCl (89.6 g/L), Na_2SO_4 (57.0 g/L), NaH_2PO_4 (88.8 g/L), $NaHCO_3$ (84.7 g/L), NaCl (175.3 g/L), urea (25.0 g/L), and 290 mg of α-amylase). The solution pH was adjusted to 6.8 using HCl 0.1 N. The mixture was added to 40 mL of water and homogenized by using a Stomacher 80 Microbiomaster (Seward, Worthing, UK) for 3 min. After this time, 0.5 g of pepsin (14,800 U) dissolved in HCl 0.1 N was added and the solution pH was adjusted to 2.0 using HCl 6 N. The solution was incubated at 37 °C in a Polymax 1040 orbital shaker (250 g) (Heidolph, Schwabach, Germany) for 2 h. Then the pH was adjusted to 6.5 using $NaHCO_3$ 0.5 N and 5 mL of a mixture of pancreatin (8.0 mg/mL) and bile salts (50.0 mg/ mL) (1:1; v/v), dissolved in 20 mL of water, were added and incubated at 37°C in an orbital shaker (250 g) for 2 h. These latter samples (representing the intestinal phase) were centrifuged at 6,000 g for 10 min and the supernatants were collected and lyophilized. One mg of lyophilized sample was firstly dissolved in 500 µl methanol, vortexed for 1 min, sonicated for 10 min, and then centrifuged (14,000 g, 10 min, 4 °C) to precipitate the salts. The supernatant was collected, appropriately diluted, and underwent HPLC analysis for the characterization of the polyphenol profile.

DPPH and ABTS Asssays

The DPPH assay was performed by adding 100 µL of each sample to 1000 µL of a methanol solution of DPPH (153 mmol L−1). The decrease in absorbance was determined with a UV-visible spectrophotometer (Beckman, Los Angeles, CA, USA) at 515 nm. The absorbance of DPPH radical without antioxidant, i.e., the control, was measured as basis. All determinations were in triplicate. Inhibition was calculated according to the formula:

$$[(Ai - Af)/Ac] \times 100$$

where Ai is absorbance of sample at t = 0, Af is the absorbance after 6min, and Ac is the absorbance of the control at time zero [1].

Trolox was used as standard antioxidant. Results were expressed in mmol Trolox Equivalent (TE).

The ABTS assay was performed according to the method described by Rufino et al. (2010) [2] with slight modifications. ABTS solution was prepared by mixing 5 mL of ABTS 7.0 mM solution and 88 µL of potassium persulfate 2.45 mM solution, which was left to react for 12 h, at 5 ◦C in the dark. Then, ethanol water was added to the solution until an absorbance value of 0.700 (0.05) at 754 nm (Beckman, Los Angeles, CA, USA). The determination of sample absorbance was accomplished at room temperature and after 6 min of reaction. All determinations were in triplicate. Inhibition was calculated according to the formula:

$$[(Ai - Af)/Ac] \times 100$$

where Ai is absorbance of sample at t = 0, Af is the absorbance after 6 min, and Ac is the absorbance of the control at time zero [2]. Trolox was used as standard antioxidant. Results were expressed TE.

Neutrophil Isolation

Blood was carefully introduced on Ficoll in a proportion of 1.5:1 and was then centrifuged at 900 g, at 4° C for 30 min. The precipitate containing the erythrocytes and neutrophils was incubated at 4° C with 0.15 M ammonium chloride to hemolyze erythrocytes. The suspension was centrifuged at 750 g, at 4° C for 15 min, and the supernatant was then discarded. The neutrophil phase at the bottom was washed first with ammonium chloride and then with phosphate buffer saline (PBS), pH 7.4 [3].

MDA Assay

Malondialdehyde (MDA) as a marker of lipid peroxidation was analyzed neutrophils by a colorimetric assay based on the reaction of MDA with a chromogenic reagent to yield a stable chromophore with maximal absorbance at 586 nm. Briefly, samples or standards were placed in glass tubes containing n-methyl-2-phenylindole (10.3 mM) in acetonitrile:methanol (3:1). HCl 12 N was added, and the samples were incubated for 1 h at 45 ◦C. The absorbance was measured at 586 nm [4].

Gene Expression

Total RNA was quantified using the Take3 Microplate in a PowerwaveXS spectrophotometer (BioTek, Winooski, VT, USA). A 1-μg sample of total RNA was reverse transcribed to cDNA using 25 U MuLV reverse transcriptase in a 5-μL retrotranscription mixture (10 mM Tris–HCl pH 9.0, 50 mM KCl, 0.1, 2.5 mM $MgCl_2$, 2.5 μM random hexamers, 10 U RNase inhibitor, and 500 μM of each dNTP) for 60 min at 42 ◦C in a Gene Amp 9700 thermal cycler (Applied Biosystems, Alcobendas, Madrid, Spain). cDNA solutions were diluted 1/10, and aliquots were frozen (−20 ◦C) until analyzed. Real-time PCR was carried out using SYBR Green technology in a LightCycler rapid thermal cycler (Roche Diagnostics, Mannheim, Germany).

References

1. Brand-Williams, W.; Cuvelier, M.E.; Berset, C. Use of free radical method to evaluate antioxidant activity. *LWT-Food Sci. Technol* **1995**, *28*, 25–30.
2. Rufino, M. do S.M.; Alves, R.E.; de Brito, E.S.; Pérez-Jiménez, J.; Saura-Calixto, F.; Mancini-Filho, J. Bioactive compounds and antioxidant capacities of 18 non-traditional tropical fruits from Brazil. *Food Chem.* **2010**, *121*, 996–1002, doi:10.1016/j.foodchem.2010.01.037.
3. Capó, X.; Martorell, M.; Sureda, A.; Tur, J.; Pons, A. Effects of docosahexaenoic supplementation and in vitro vitamin C on the oxidative and inflammatory neutrophil response to activation. *Oxid Med Cell Longev* **2015**, *2015*, 187849, doi:10.1155/2015/187849.
4. Annunziata, G.; Jimenez-García, M.; Tejada, S.; Moranta, D.; Arnone, A.; Ciampaglia, R.; Tenore, G.C.; Sureda, A.; Novellino, E.; Capó, X. Grape polyphenols ameliorate muscle decline reducing oxidative stress and oxidative damage in aged rats. *Nutrients* **2020**, *12*, 1280, doi:10.3390/nu12051280.
